# Deep brain optical coherence tomography angiography in mice: *in vivo*, noninvasive imaging of hippocampal formation

**DOI:** 10.1038/s41598-018-29975-6

**Published:** 2018-08-02

**Authors:** Kwan Seob Park, Jun Geun Shin, Muhammad Mohsin Qureshi, Euiheon Chung, Tae Joong Eom

**Affiliations:** 1 Advanced Photonics Research Institute, Gwangju Institute of Science and Technology, 123 Cheomdan-gwagiro, Buk-gu, Gwangju 61005 South Korea; 2 Department of Biomedical Science and Engineering, Gwangju Institute of Science and Technology, 123 Cheomdan-gwagiro, Buk-gu, Gwangju 61005 South Korea; 3 School of Mechanical Engineering, Gwangju Institute of Science and Technology, 123 Cheomdan-gwagiro, Buk-gu, Gwangju 61005 South Korea

## Abstract

The hippocampus is associated with memory and navigation, and the rodent hippocampus provides a useful model system for studying neurophysiology such as neural plasticity. Vascular changes at this site are closely related to brain diseases, such as Alzheimer’s disease, dementia, and epilepsy. Vascular imaging around the hippocampus in mice may help to further elucidate the mechanisms underlying these diseases. Optical coherence tomography angiography (OCTA) is an emerging technology that can provide label-free blood flow information. As the hippocampus is a deep structure in the mouse brain, direct *in vivo* visualisation of the vascular network using OCTA and other microscopic imaging modalities has been challenging. Imaging of blood vessels in the hippocampus has been performed using multiphoton microscopy; however, labelling with fluorescence probes is necessary when using this technique. Here, we report the use of label-free and noninvasive microvascular imaging in the hippocampal formation of mice using a 1.7-μm swept-source OCT system. The imaging results demonstrate that the proposed system can visualise blood flow at different locations of the hippocampus corresponding with deep brain areas.

## Introduction

The hippocampus is part of the limbic system in the brain, and plays an important role in transmitting signals to other parts of the brain^1^. It is associated with learning and memory, and regulating emotions^2^. In addition, it plays a role in regulating the function of the hypothalamus^2^. Therefore, hippocampal disorders are closely related to brain diseases, such as Alzheimer’s disease, dementia, and epilepsy^2^.

Blood vessels transmit nutrients to the whole body. Capillaries are the smallest blood vessels. They are the site of gas and nutrient transport between the bloodstream and tissues in the body, and are directly or indirectly related to abnormal function in organs^3,4^. Morphological changes in capillaries within the hippocampal formation have been observed in a mammalian aging study^3^. In addition, changes in the density and thickness of capillaries in the hippocampal formation occur first in the early stages of Alzheimer’s disease^4^. Cerebral blood flow has also been closely linked to Alzheimer’s disease. Thus, microvascular information, such as the vascular network and blood flow of the hippocampal formation, is an indicator of the onset or progression of certain brain diseases. Therefore, observing the microvasculature of the hippocampus will be helpful in identifying the aetiology of diseases and drug development for those brain diseases.

An imaging depth of at least 1 mm is necessary to visualise the microvascular network in the hippocampal formation of mice; however, it is difficult to directly access this depth with the current microscopic imaging systems^5^. Therefore, several attempts have been made to increase the penetration depth of microscopy. Previously, the hippocampal microvasculature and neurons have been visualised using two photon microscopy^6^. Visualisation was performed by inserting a glass capillary with a glass window through the cortex to the hippocampus to observe hippocampal neurons and microvasculature directly using an endoscope. Recently, noninvasive and *in vivo* imaging of neurons and blood vessels in the mouse brain, of up to approximately 2 mm in depth, has been achieved using three-photon microscopy^7^. A fluorescence signal is only generated at the focal spot during imaging; therefore, a multiphoton microscope can maximise the image contrast, resulting in increases in imaging depth. However, these approaches utilise fluorescence dye to obtain sufficient contrast, which increases the complexity of the experimental preparation procedure. Furthermore, the penetration depth of the multiphoton microscopy remains insufficient to visualise the vascular network of the hippocampal formation in mice^7^.

Recently, optical coherence tomography (OCT) has been applied to visualise the hippocampal formation in the mouse brain. The usefulness of OCT has been successfully demonstrated in a variety of applications based on its ability to noninvasively acquire three-dimensional (3D) volumetric data with high resolution (1–15 µm) in real time^8^. The development of optical coherence tomography angiography (OCTA) provided functionality to OCT and its applications. OCTA is a technique that extracts dynamic scattered signals, induced by moving blood cells, from volumetric OCT data^9^. OCTA utilises intrinsic contrast to acquire a blood vessel image; therefore, a contrast agent is not required.

OCT has limitations in penetration depth similar to other microscopic imaging modalities. In 2015, *in vivo* vascular imaging of the subcortical mouse brain with a 1.7-μm spectral-domain OCT system (SD-OCT) was reported, which demonstrated that the longer wavelength of the OCT light source penetrates deeper into the mouse brain^10^. The vascular network could be visualised up to near the corpus callosum (CC). The SD-OCT system had system sensitivity roll-off of approximately 10 dB at 2.5 mm. In 2016, a 1.3-μm SS-OCT system using the vertical-cavity surface-emitting laser was proposed for use in deep brain microvascular imaging^11^. This study performed optical coherence Doppler tomography using this system, and reported the observation of blood vessels at the cornus ammonis (CA) region of hippocampal formation; however, an image of the vascular network was not provided.

In this study, we demonstrated the feasibility of label-free, noninvasive deep brain vascular imaging using *en face* maximum intensity projection (MIP) images acquired by OCTA with 1.7-µm swept laser source in mice. The reduction of light scattering with the longer wavelength source enhances the penetration depth of the OCT probe beam in brain tissue. Moreover, the narrow spectral linewidth of the 1.7-µm swept-source laser improves the capable imaging depth of the OCT system. We compared the image contrast of 1.7-μm OCTA with 1.3-μm OCTA, which is the conventional approach for mouse brain imaging. Multiple depth focusing has been applied to prevent blurring and invisibility in angiography when the imaging plane is out of the lens’ depth of field (DOF). We visualised the segmented vasculature for each hippocampal region from top to bottom by changing focal depth without losing resolution.

## Results

### 1.7-µm swept-source laser

**Figure 1:**
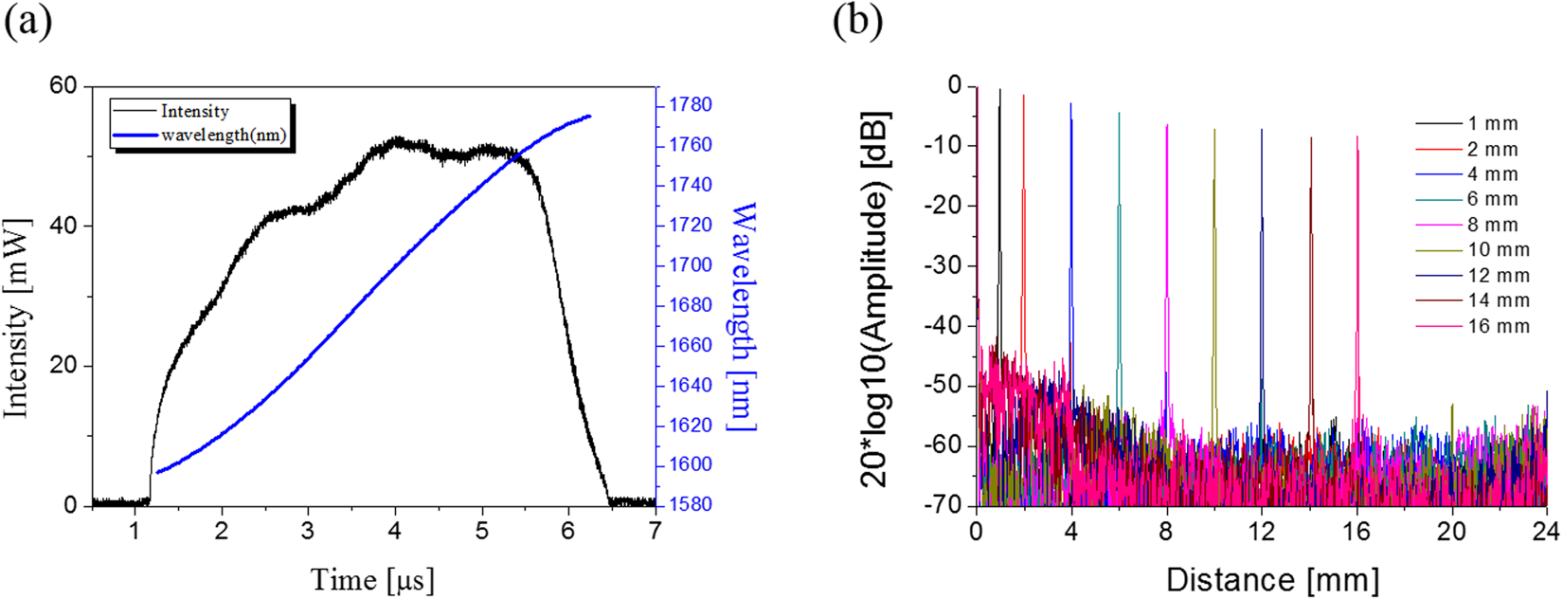
Characteristic of 1.7-µm swept-source laser. (**a**) Spectral characteristics of the swept-source. (**b**) Sensitivity roll-off characteristic of the system with the swept-source. A roll-off characteristic of the laser represents the sensitivity drop less than 2 dB at a 4 mm in depth.

To ensure a high penetration depth of the probe beam, we selected the 1.7-μm swept-source laser, which has a longer wavelength band than the 1.3-μm swept-source laser, which is most common in the SS-OCT system. The spectral characteristic of the source used in the experiments showed that the centre wavelength of the source was approximately 1.7 µm and ranged from 1600–1790 nm (Fig. 1(a)). The sweeping rate of the source was 90 kHz. To measure the roll-off characteristic of the laser, we configured a Michelson interferometer and measured intensity drop while moving a reference mirror up to 16 mm. Figure 1(b) shows this result, representing a sensitivity drop < 2 dB at a depth of 4 mm.

### *In vivo*, label-free blood flow imaging in the mouse hippocampal formation

**Figure 2:**
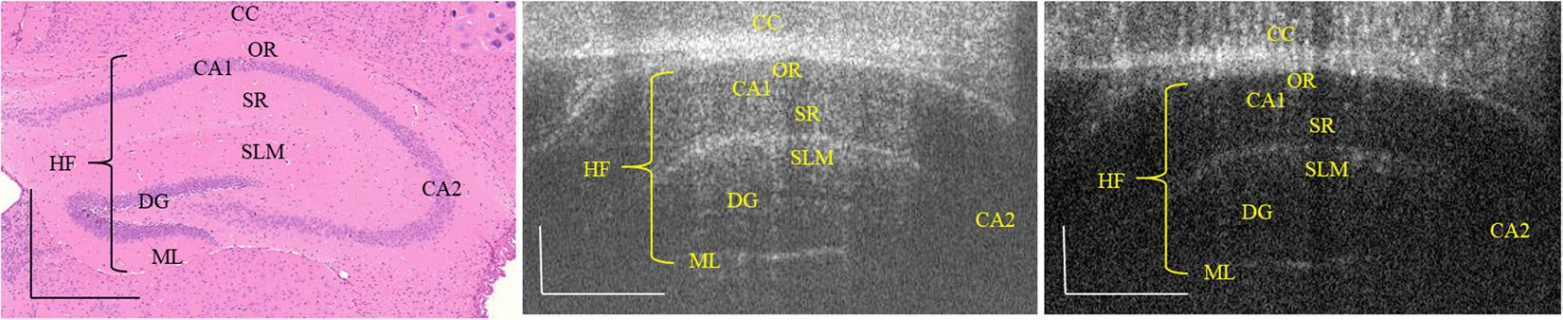
From the left, H&E histology showing the hippocampal formation, corresponding OCT image and OCTA image. CC, corpus callosum; OR, stratum oriens; CA, cornu ammonis; SR, stratum radiatum; SLM, stratum lacunosum-moleculare; DG, dentate gyrus; ML, molecular layer. The scale bar represents 500 µm.

**Figure 3:**
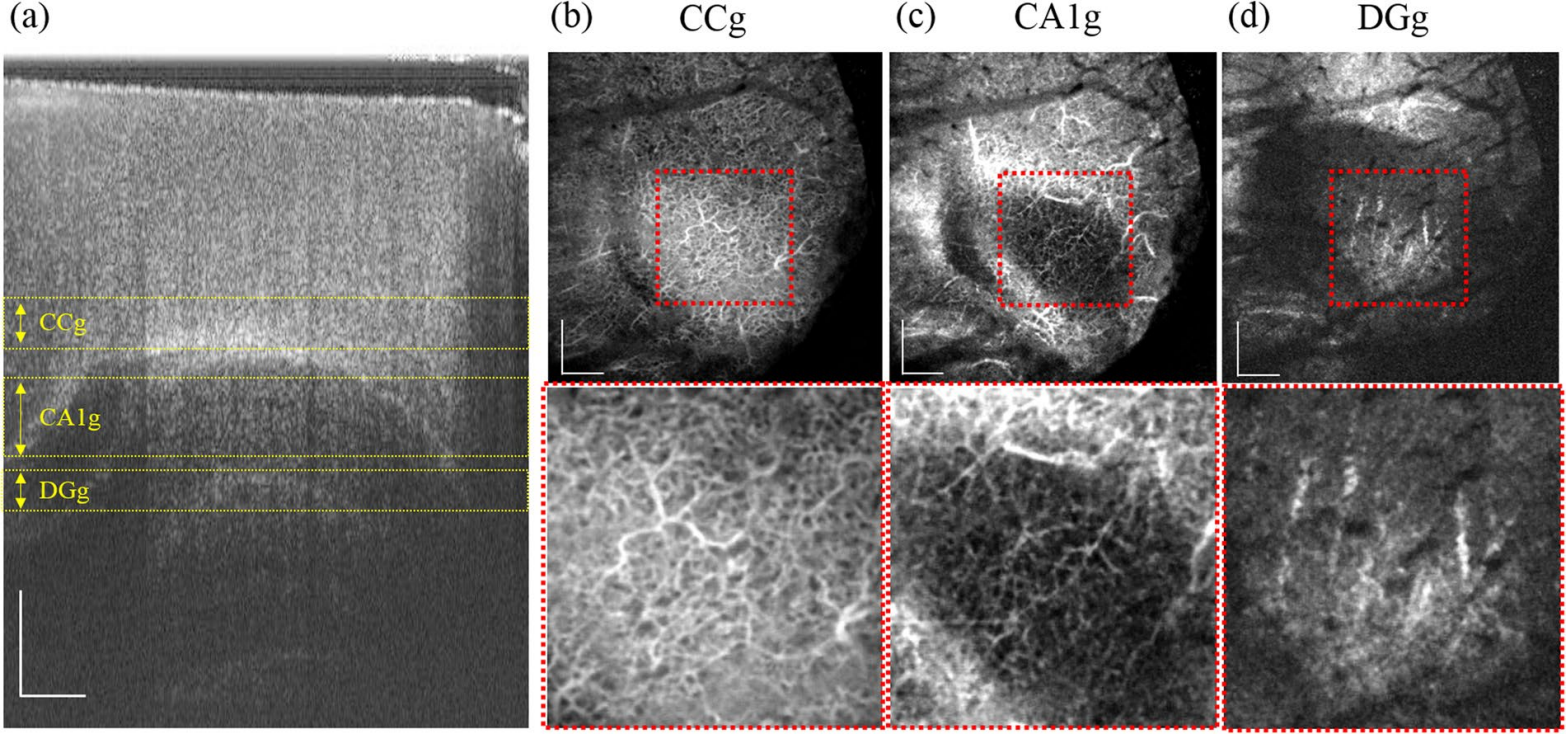
*In vivo* imaging of the structure and microvasculature in the hippocampal formation of a mouse brain. (**a**) OCT structure image. (**b**–**d**) *en face* MIP of vasculature at the depth of CCg (800–1120 µm), CA1g (1290–1600 µm), and DGg (1730–1900 µm) indicated in (**a**), respectively. Zoomed images representing the region of white rectangle in (**b**–**d**) images were shown below. CCg: deep cortex and corpus callosum, DGg: stratum lacunosum-moleculare. The scale bar represents 500 µm.

We have imaged the mouse hippocampal formation using a developed 1.7-µm SS-OCT system. In this experiment, the brain of a 5-week-old mouse was imaged to observe the hippocampal vasculature through a cranial window. To confirm the histological position of the obtained OCT brain images, we compared the OCT structure image and OCTA image with H&E histology (Fig. 2). The OCTA image was extracted by applying a speckle variance algorithm to separate dynamic signals due to blood cell movement from the 3D data set. A 3D rendered OCTA movie was provided as Supplementary Video S1. Following this, we produced *en face* MIP images using the volumetric OCTA data to show the connectivity of the vessels at each depth. Each range (CCg, CA1g, and DGg in Fig. 3) covered a depth of 795–1123 µm, 1293–1600 µm, and 1727–1897 µm, respectively. Figure 3(a) shows a cross-sectional OCT image and Fig. 3(b–d) represent MIP images corresponding to each region, as indicated in Fig. 3(a). The oval-shaped shadow is due to differences between the inner (hippocampus) and outer (corpus callosum) regions. The enlarged view within the dotted rectangle shows the detailed vessel network, which reveals different vascular network patterns in each region^12^. Figure 3(b) represents the blood vessels near the CC. Figure 3(c) includes the CA1 region, which plays an important role in the matching and mismatching of information obtained from the CA3 region^2^. Figure 3(d) contains the stratum lacunosum-moleculare layer of the DG, which is surrounded by the CA and plays a crucial role in processing information from the entorhinal cortex to the CA3^13^.

### Comparison of 1.7- and 1.3-μm OCTA

**Figure 4:**
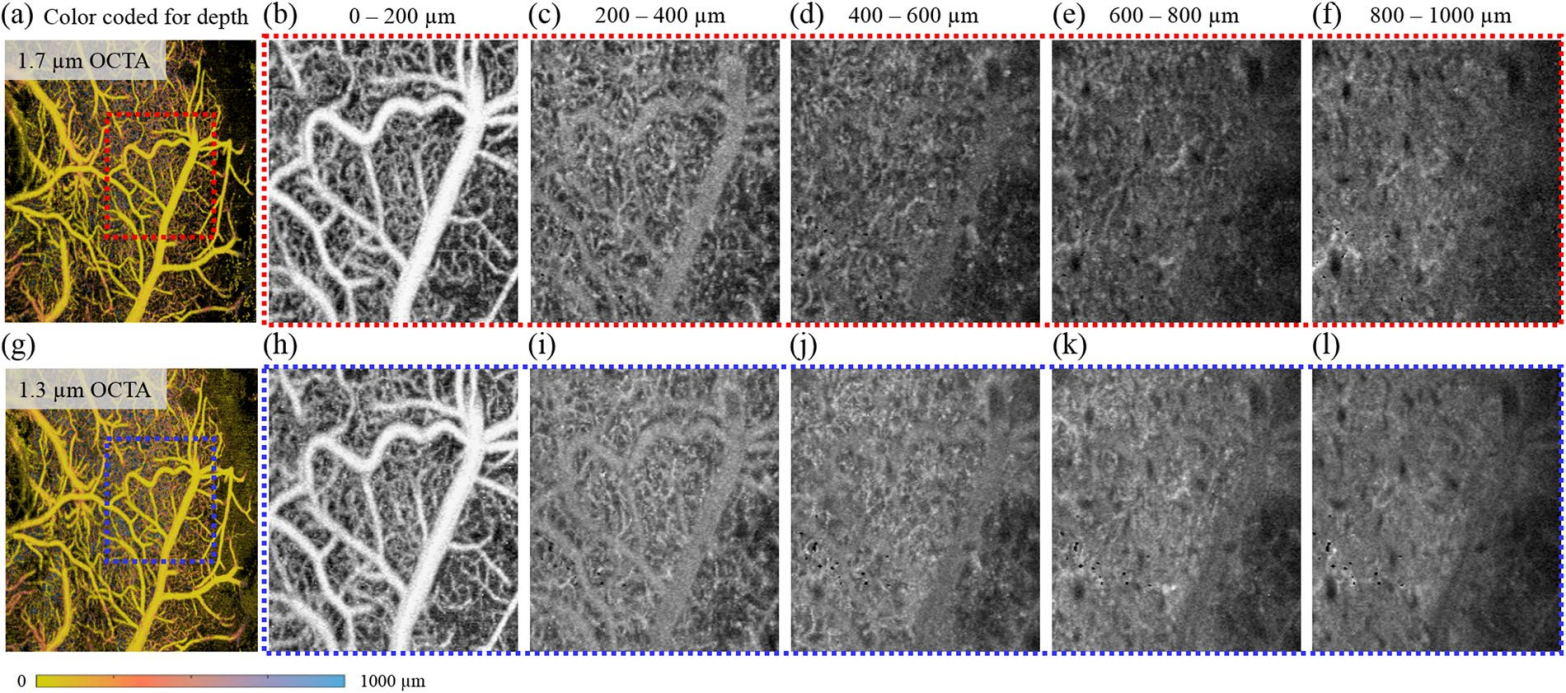
Image quality comparison of 1.7 µm and 1.3 µm OCTA. (**a**–**f**) MIP images from 1.7-µm OCTA. (**g**–**l**) MIP images from 1.3-µm OCTA. (**a**,**g**) Colour-coded MIP images at all depths. (**b**–**f**,**h**–**l**) Gray-scaled MIP images at certain depths enlarged in the red and blue dotted square of (**a**) and (**g**), respectively.

To confirm performance of the 1.7-μm OCTA with deep brain imaging, we have compared 1.7- and 1.3-μm OCTA imaging. To ensure comparable conditions, the sensitivity of the 1.3-μm OCT system was carefully controlled by adjusting the reference power. Both systems shared the probe arm and imaged the same location. Following this, a 3D data set was acquired using 2D-glavo scanning while focusing the probe beam at the brain surface. We expressed the 3D data using *en face* MIP images to represent the results (Fig. 4). Prior to projection of the OCTA image, the volumetric OCTA signals were mapped using 1% and 99% saturated at low and high intensity histograms, respectively, of each OCTA image to match the contrast conditions. Colour-coded MIP images for two volumetric OCTA datasets are shown in Fig. 4(a,g). Figure 4(b–f,h–l) are grayscale MIP images of certain depth bands, which is zoomed from the areas in the red and blue dotted squares (Fig. 4(a,g)). The shallow vessels (Fig. 4(b,h)) appear to be similar; however, 1.7-µm OCTA images retain a better contrast despite low brightness at increased depths, while 1.3-µm OCTA images showed higher brightness but lower contrast. To further demonstrate the differences between 1.7- and 1.3-µm OCTA images, we followed the line profiles at different depth positions in cross-sectional OCTA images and analysed each profile. We found that the 1.7-µm OCTA images showed superior contrast even at deep locations (Supplementary Fig. S1).

### *En face* visualisation of blood vasculature acquired at multiple focus locations

**Figure 5:**
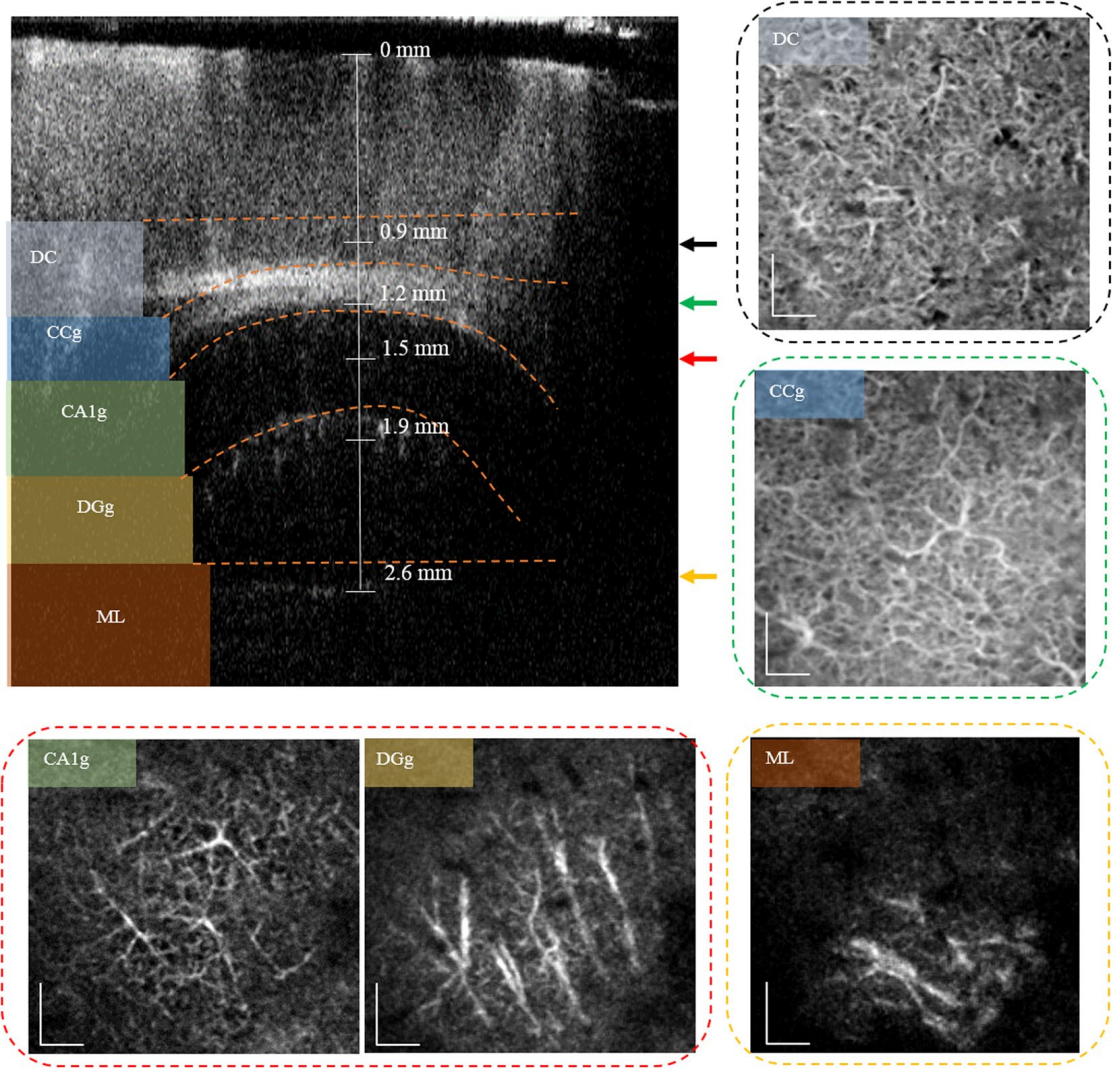
Total merged OCTA with 4 different focal depths and the segmented *en face* mapping of the blood vessels in the deep region of the mouse brain. The cross-section image acquired on the focal plane of the probe lens located at the position indicated by the orange arrow. Each layer was segmented manually along the orange dotted line in the cross-section image to remove unwanted signals. The DC layer shows blood vessels near the deep cortex, which is obtained on the focal plane of the probe lens shown by the black arrow. The CC layer shows blood vessels in the corpus callosum and alveus of the hippocampus. The CA1g and DGg was acquired on the focal plane at the position indicated by the red arrow, which visualised the blood vessels in the CA1 region of the hippocampal formation and near dentate gyrus (DG). ML shows the blood vessels in the molecular layer surrounding the DG. The scale bar represents 200 µm.

The calculated DOF of the lens used in experiments was 1.8 mm in air, which can cover the whole hippocampal formation (~1.4 mm in air). However, OCTA images of the whole hippocampal formation could not be obtained using a single imaging. It is believed that the effective DOF is slightly reduced due to the turbidity of mouse brain tissue. Blood vessels near the hippocampus are a few microns in size; therefore, they will be easily blurred or invisible when the imaging plane is not in line with the focal plane. Four different data sets were obtained while changing the focal position along the axial direction to correct for this. Each focal position is indicated by different coloured arrows (Fig. 5). Following this, blood flow signals of the focused region were *en face* mapped from each 3D data set, providing good contrast images from 0.9–2.6 mm in depth (Fig. 5). We observed vessel connectivity to a depth of 2.6 mm. The hippocampus is curved in shape; therefore, the respective layers were visualised by manually segmenting the position that was indicated by the orange dotted line. The deepest *en face* OCTA visualised the vessel network in the molecular layer at bottom of hippocampal formation. In addition, we have combined several 3D data sets acquired at different focal positions to reconstruct 3D OCTA data sets with optimal resolution by realigning and applying different weighted values into the whole data set. We have provided a reconstructed 3D OCTA movie that shows the *in vivo* deep brain vessel structure (Supplementary Video S2) in a mouse model study. This video clearly shows the microvasculature of the hippocampal region (CA1 and DG).

## Discussion

The proposed OCTA system used the wavelength swept laser with a long coherence length (>11 mm), which contributed to greater roll-off characteristics (2 dB falling-off at 4 mm) when compared with previous 1.7-μm SD-OCT systems^10^. The better sensitivity roll-off characteristic of the SS-OCT system enabled deeper OCT angiography in the mouse brain with high contrast.

In samples with >70% of the water content, the total attenuation coefficient has been reported as 1.8 and 1.45 in 1.3 and 1.7 μm wavelength bands, respectively^14^. The 1.7-μm OCT is able image deeper areas in phantom and biological samples, such as human fingertips, excised human teeth, rubber, and 10% intralipids. Other studies have shown a higher signal to noise ratio (SNR) in the 1.7-μm OCT system when compared with the 1.3-μm OCT system at ~600 μm into the brain tissue^15^. These previous studies imply characteristic scattering dominantly contributes to the longer penetration depth of the light more than the absorption characteristic. In addition, the relatively high scattering coefficient of 1.3-μm wavelength increases multiple scattering in OCT signals, which lowers the image contrast by increasing the background noise of the OCT image. Our comparison study showed that the effect of multiple scattering along the wavelength is apparent due to differences in the background level in the deep regions of the cross-sectional OCTA image. Furthermore, *in vivo* OCTA imaging inevitably involves movement of the sample (for example, respiratory and cardiac motion), which additively increases background noise level *via* speckle noise from auto-correlated interference signals. Thus, speckle noise due to multiple scattering with wavelength dependency may be reduced when using a 1.7-μm source. The deeper the region, the greater the contribution of multiple scattering^14^. Therefore, the lower scattering coefficient in the 1.7-μm wavelength provided a clearer angiographic image, which was favourable for deep brain angiography.

There are improvements that can be made to the 1.7-μm SS-OCT for the application of deep brain imaging. First, in this study, optical components, such as collimators and optical fibre circulators in the interferometer, were not optimised for 1.7-μm the wavelength bandwidth due to a lack of provider. The sensitivity of the system would be maximised if these components were replaced with equipment calibrated to 1.7-μm OCT. Second, a full-range imaging method with detection of the quadrature signal or frequency shift might be useful to further improve the imaging depth by retaining a higher SNR at greater depths^16,17^.

In summary, we have described an *in vivo*, label-free observation of the hippocampal vascular network in the mouse brain using the 1.7-μm SS-OCT angiography system. This system can image up to the bottom of the hippocampal formation. We have reported the high sensitivity and roll-off characteristics of the 1.7-μm SS-OCT system, therefore achieving noninvasive, label-free, *in vivo* deep vascular imaging. Furthermore, we found that a lower optical scattering characteristic of 1.7-μm was useful for deep brain OCTA imaging due to lower background levels in the image when compared with the 1.3-µm wavelength light. Taken together, we have shown that 1.7-μm SS-OCT angiography can provide images up to the depth of the hippocampal formation with high spatial and temporal resolution. Deep brain angiography will be beneficial to understand several pathologies, such as Alzheimer’s disease, dementia, and epilepsy.

## Methods

### 1.7-µm Optical coherence tomography system

We utilised an SS-OCT system to *in vivo* image the structure and microvasculature of the mouse brain (Supplementary Fig. S2). The swept laser (HSL-40, Santec, Inc.) used in all experiments had a central wavelength and bandwidth of 1.68 µm and >150 nm, respectively, and operated with a sweeping rate of 90 kHz (Fig. 1). The light source was divided by a fibre coupler (10/90) into two paths, the reference and sample arms. In the reference arm, light propagated through a circulator and was reflected at a reference mirror. In the sample arm, the light also propagated through a circulator and 2D galvanometric scanner and was back-scattered from a sample. These two lights were interfered at a fibre coupler (50/50) that was installed in front of a balanced photodetector (BPD-200, Santec, Inc.). Next, the interfered signal was quantised by a digitiser (ATS9350, Alazartech, Inc.). The 2D galvanometric scanner was controlled by the signal from a function generator and synchronised with the swept-source and digitiser. The full width of half maximum of the spectrum exceeded 100 nm, and the corresponding axial resolution was 15 µm. The measured axial resolution was degraded to 25 µm. This decrease could be due to the limited data that was acquired by performing a k-clock, and the limited operating wavelength of the circulator. The lateral resolution of the system was 28 µm when using a lens with a focal length of 30 mm. The sample was irradiated with an average optical power of 7 mW during image acquisition.

### 1.3-µm Optical coherence tomography system

We configured the OCT system operating at a central wavelength of 1.3 µm to compare with the OCTA performance as a function of wavelength. The optical arrangement for an interferometer was identical with the 1.7-µm OCT system. The bandwidth and sweeping rate of the light source (1310 nm, SSOCT, Axsun, Inc.) was 110 nm and 100 kHz, respectively. The same galvanometric scanner was used in the 1.7-µm and 1.3-µm OCT systems to acquire images in a same field of view. In addition, the 1.3-µm OCT system used the same k-clock signals as the 1.7-µm OCT system for data acquisition.

### K-clock and digital dispersion compensation

A spectral interferogram, detected using the balanced detector, was sampled at 1024 points per A-scan *via* the digitiser for data acquisition in both 1.7-µm and 1.3-µm OCT systems. A k-clock signal was generated using an additional auxiliary interferometer as a wavenumber linear sampling clock. Thus, cross-sectional OCT images were obtained using Fast Fourier Transformation (FFT) of the interferogram. Numerical dispersion compensation was performed on all A-line data prior to the FFT to remove dispersion at each A-line depth^18^.

### Image acquisition and optical microangiography

Volumetric OCT data was acquired at a line rate of 90 kHz in a 4 × 3 mm field of view using raster scanning provided by 2D galvanometric scanners. Each scan took 27 seconds. In the slow axis C-scan, each B-frame contained 500 A-lines, with a total of 4000 B-frames acquired with 8 repetitions at each location. Next, a speckle variance-based OCTA was used to visualise volumetric blood vessels at the capillary level^19^.

### Animal preparation

All the animal handling protocols followed the guidelines of the Institutional Animal Care and Use Committee (IACUC) at the Gwangju Institute of the Science and Technology (GIST), Korea. All experimental protocols were approved by the GIST IACUC under protocol #GIST-2016–11. We used two immunodeficient male mice, 4–6 weeks old with body weight between 18–23 g. Mice were anaesthetised with a zoletil/xylazine mixture in saline solution (60/10 mg/kg). The body temperature was maintained at 37 °C. The craniotomy was performed according to the description from R. Mostany and colleagues^20^. Briefly, the diameter of the drilling site was approximately 6 mm, and the cover slip over the exposed brain was 8 mm. The cover slip was fixed with dental acrylic and cyanoacrylate glue. To stabilise the animal on the heating pad we used a customised angel ring (Customised ring type *in-vivo* heating system, Live Cell Instrument, South Korea)^21^.

### Data processing for image merging

Volumetric OCTA data with optimal resolution along the whole depth was obtained by merging five OCTA volume data sets acquired while changing the axial focal position of the sample at equidistant points. Prior to merging the volumes, rigid volume registration, including rotation and translation, were performed on the OCTA data to normalise all samples in the same axial and lateral locations. Following this, spatially aligned volumes were locally weighted along the depth of the Gaussian window and merged using the following equation:

$${I}_{merged}=({I}_{1}{W}_{1})+({I}_{2}{W}_{2})+({I}_{3}{W}_{3})+({I}_{4}{W}_{4})+({I}_{5}{W}_{5})$$

where ${I}_{{merged}}$, ${I}_{n},$ and ${W}_{n}$ are merged, aligned volumes, and weights, respectively. We selected the focal position of each volume as the central position of the Gaussian window. The sum of all Gaussian weights was designed to maintain the multiplicative identity of the intensity. The merged volumetric data sets provided clear OCTA images in deep brain areas with uniform resolution and intensity.

## Electronic supplementary material

Supplementary information

Supplementary Video S1

Supplementary Video S2
