# Supplementary material for: Deep brain optical coherence tomography angiography in mice: in vivo, noninvasive imaging of hippocampal formation
Source: Sci Rep. 2018 Aug 2;8:11614. doi: 10.1038/s41598-018-29975-6 (PMC6072748; doi:10.1038/s41598-018-29975-6)
Supplement: Supplementary file 1 — Supplementary information [file 41598_2018_29975_MOESM1_ESM.docx]

Deep brain optical coherence tomography angiography in mice: *In vivo*, noninvasive imaging of hippocampal formation

Kwan Seob Park^1^, Jun Geun shin^1^, Muhammad Mohsin Qureshi^2^, Euiheon Chung^2,3^, Tae Joong Eom^1,*^

^1^Advanced Photonics Research Institute, Gwangju Institute of Science and Technology, 123 Cheomdan-gwagiro, Buk-gu, Gwangju 61005, South Korea

^2^Department of Biomedical Science and Engineering, Gwangju Institute of Science and Technology, 123 Cheomdan-gwagiro, Buk-gu, Gwangju 61005, South Korea

^3^School of Mechanical Engineering, Gwangju Institute of Science and Technology, 123 Cheomdan-gwagiro, Buk-gu, Gwangju 61005, South Korea

^*^Correspondence and requests for materials should be addressed to T.J.E. ([eomtj@gist.ac.kr](mailto:eomtj@gist.ac.kr)).

**Supplementary Notes**

In order to obtain quantitative numbers, such as mean SNR or CNR, it is necessary to set the region of interest. We drew a line profile with image processing by flattening the image to get intensity values at the same depth. Following this, the standard deviations of intensity values on the line profiles were obtained and plotted as a function of depth in the Supplementary Figure S1.

### Root-mean-square (rms) contrast

The contrast of each image can be defined by RMS contrast ^1^. This RMS contrast does not depend on spatial frequency content of the image or the spatial distribution of contrast in the image. The RMS contrast, denoted by *rms*, is known as the standard deviation of pixel intensities, and is defined as follows:

$rms=\left[\frac{1}{n-1}\sum_{i=1}^{n} (x_{i}-\bar{x})^{2}\right]^{1/2},$ (1)

where $x_{i}$ is a gray-level pixel value and $\bar{x}$ is the mean gray-level pixel value.

**Supplementary Reference**

1. Peli, E. Contrast in complex images. *J. Opt. Soc. Am. A* **7,** 2032-2040 (1990).

**Supplementary Figures**

**
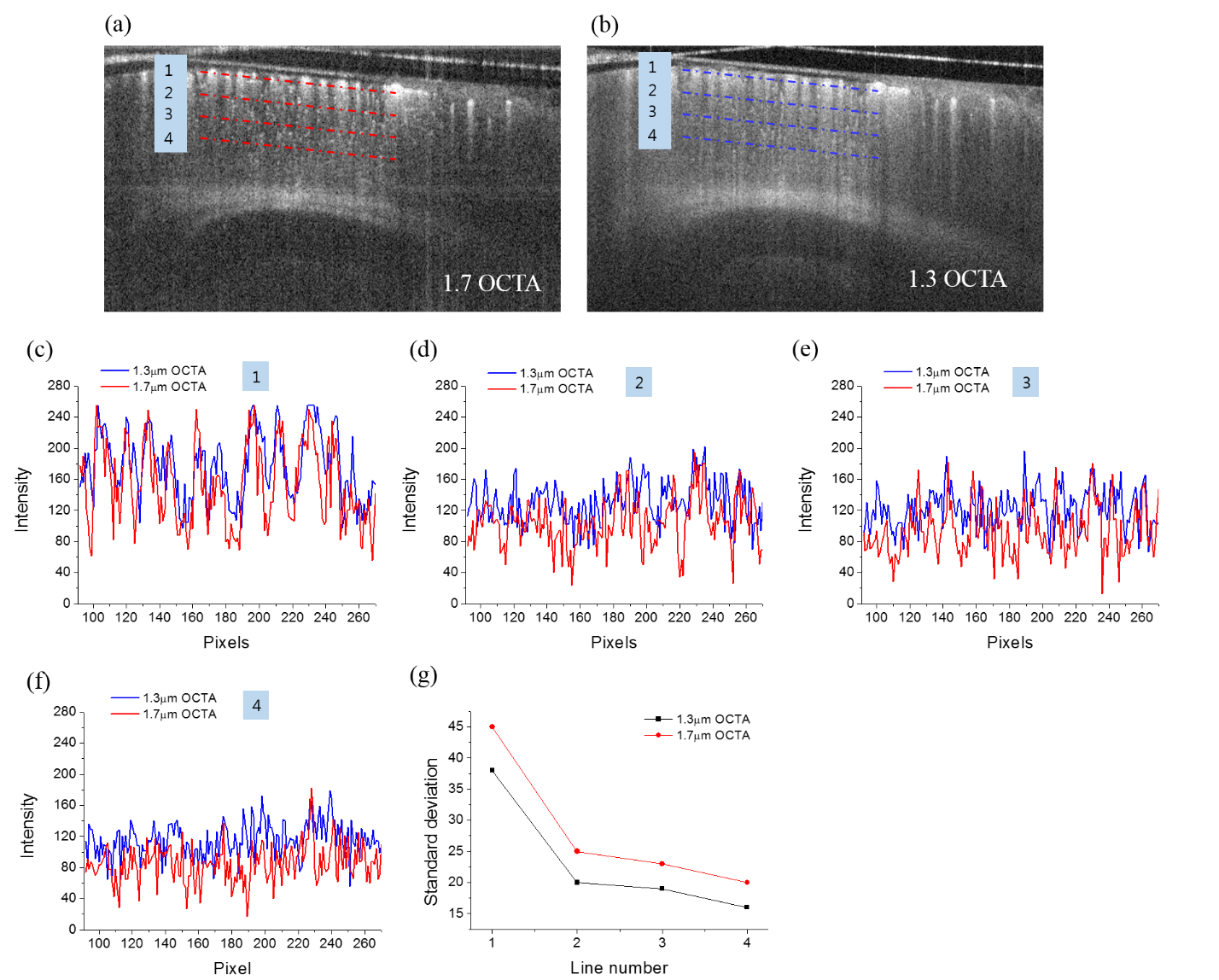
**

**Supplementary Figure S1 | Comparison of 1.7- and 1.3-µm OCTA.**

1.7-µm (a) and 1.3-µm (b) cross-sectional OCTA images. (c-f) Intensity profiles at the red and blue dotted lines of (a) and (b). (g) Standard deviation of intensity values on the lines in the each OCTA image as a function of depth (100µm, 250 µm, 300 µm and 450 µm from the bottom surface of the glass window). The intensity profiles were averaged as three pixels along the depth to remove speckle error.

**
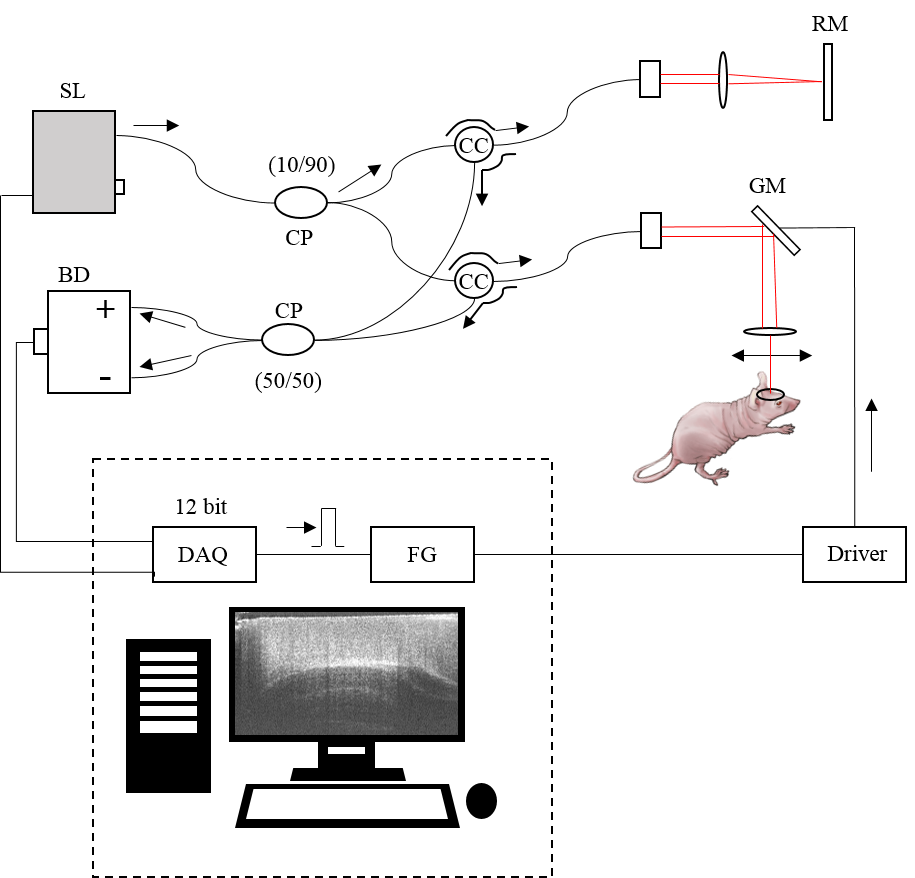
**

**Supplementary Figure S2 | Illustration of experimental setup.**

SL: swept-laser, BD: balanced detector, CP: coupler, CC: circulator, RM: reference mirror, GM: galvanometric mirror, DAQ: digital acquisition card, FG: function generator.

**Supplementary Movies**

**
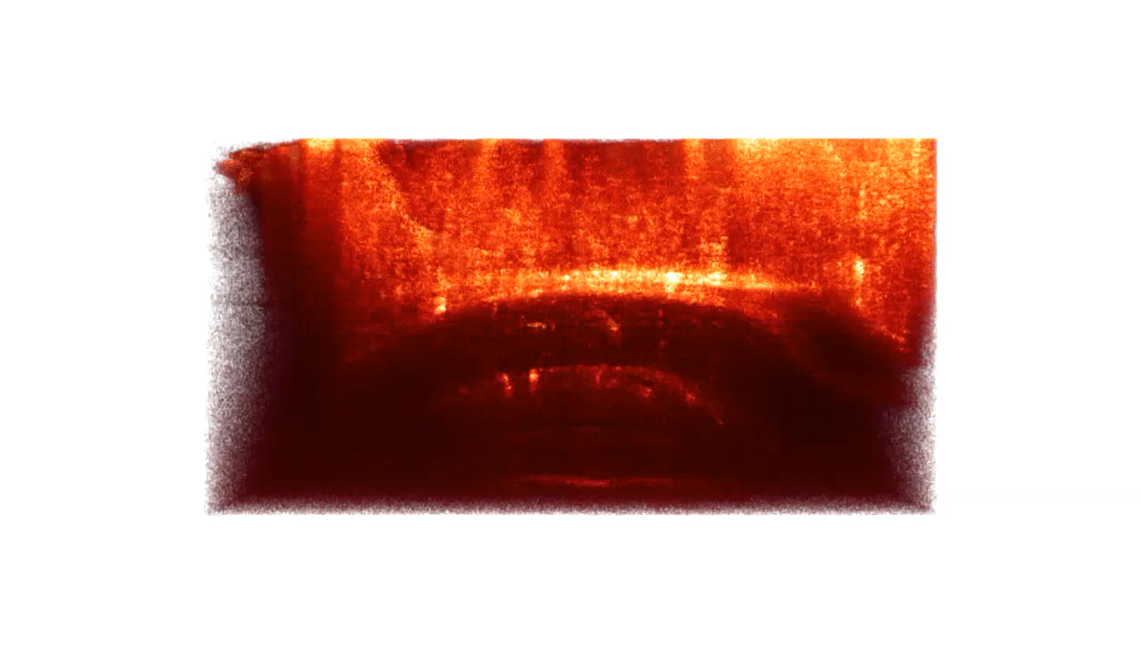
**

**Supplementary Video S1 | 3D rendered mouse brain OCTA image movie acquired by the 1.7µm SS-OCT system. The movie was processed by commercial software, Amira.**

**
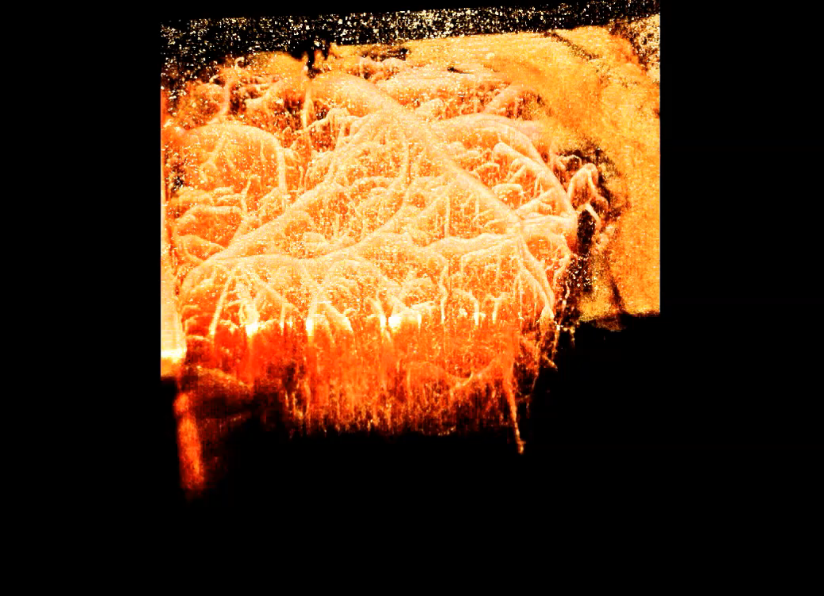
**

**Supplementary Video S2 | 3D rendered OCTA image movie using the merged data. The movie was processed by commercial software, Arima.**
